# Joint transcriptomic and metabolomic analysis reveals the mechanism of low-temperature tolerance in *Hosta ventricosa*

**DOI:** 10.1371/journal.pone.0259455

**Published:** 2021-11-03

**Authors:** QianQian Zhuang, Shaopeng Chen, ZhiXin Jua, Yue Yao

**Affiliations:** College of Agriculture, Jilin Agricultural Science and Technology University, Jilin, PR China; National Institute of Plant Genome Research, INDIA

## Abstract

*Hosta ventricosa* is a robust ornamental perennial plant that can tolerate low temperatures, and which is widely used in urban landscaping design in Northeast China. However, the mechanism of cold-stress tolerance in this species is unclear. A combination of transcriptomic and metabolomic analysis was used to explore the mechanism of low-temperature tolerance in *H*. *ventricosa*. A total of 12 059 differentially expressed genes and 131 differentially expressed metabolites were obtained, which were mainly concentrated in the signal transduction and phenylpropanoid metabolic pathways. In the process of low-temperature signal transduction, possibly by transmitting Ca^2+^ inside and outside the cell through the ion channels on the three cell membranes of COLD, CNGCs and CRLK, *H*. *ventricosa* senses temperature changes and stimulates SCRM to combine with DREB through the MAPK signal pathway and Ca^2+^ signal sensors such as CBL, thus strengthening its low-temperature resistance. The pathways of phenylpropanoid and flavonoid metabolism represent the main mechanism of low-temperature tolerance in this species. The plant protects itself from low-temperature damage by increasing its content of genistein, scopolentin and scopolin. It is speculated that *H*. *ventricosa* can also adjust the content ratio of sinapyl alcohol and coniferyl alcohol and thereby alter the morphological structure of its cell walls and so increase its resistance to low temperatures.When subjected to low-temperature stress, *H*. *ventricosa* perceives temperature changes via COLD, CNGCs and CRLK, and protection from low-temperature damage is achieved by an increase in the levels of genistein, scopolentin and scopolin through the pathways of phenylpropanoid biosynthesis and flavonoid biosynthesis.

## Introduction

Environmental factors such as temperature, humidity and light intensity limit the geographical distribution of plants [1]. However, studies have shown that it is not photosynthesis but temperature that limits the growth range of plants [2]. In the process of introducing garden plants in different areas for urban landscaping and ecological construction, although most exotic garden plants can only thrive under standardized management regimes, studies have shown that climate adaptability is positively correlated with the success of domestication [3]. Among the many climatic factors, it is temperature that has the greatest effect on plant growth and development [4]. Jilin Province in China is located in a mid- to high-latitude region, and has a typical temperate continental monsoon climate, with long cold winters and extremely low temperatures. From 1961 to 2010, the average number of cold hazards in Jilin Province was three per decade [5], and these extremely low temperatures have severely restricted the introduction and domestication of ornamental plants in Northeast China.

Temperature stress in plants has been extensively studied. Very high temperatures in summer have the greatest impact on plant productivity, but low temperatures in winter affect the survival of species [4]. Low temperatures can trigger complex changes in plant morphology, physiology and biochemistry [6], including cold signal transduction, and changes in reactive oxygen species (ROS) and cold-induced gene transcription products [7]. When a cold-resistant variety of *Brassica napus* is subjected to a low-temperature environment for long periods, the initial plant response involves activation of the osmotic mediation system and triggering of the antioxidant enzymes. Transcriptome analysis has shown that rapeseed protein serine/threonine kinase, inositol-1-phosphate synthase and calmodulin all play an important role in low-temperature stress [8]. In the cold-stress signal transduction pathway of *Arabidopsis thaliana*, the histidine kinases *AHK2* and *AHK3* are involved in the recognition of low-temperature (1°C) induction in response to the expression of the A-type Arabidopsis response regulator (*ARR*) gene family. The *CBF* family is also involved in important cold-signal transduction pathways in *Arabidopsis* [9]. Flavonoids are the main determinants of cold-stress response. Under low-temperature conditions, *Arabidopsis* increases the expression of flavonoid synthesis genes, thereby increasing the flavonoid content in response to low-temperature stress [10]. *Hordeum distichon* (two-rowed barley) is a crop that is cultivated on the Qinghai-Tibet Plateau. After cold-stress treatment, eight metabolites of sensitive and tolerant types change, such as deoxyadenosine and 6-methylmercaptopurine [11]. There have also been many studies of the effects of cold stress on the model plant *Nicotiana tabacum*. In a study of the transcriptomes and metabolites produced by the two ecotypes of *Nicotiana tabacum*, namely *CB*-*1* and *K326*, it was found that there was an approximately 50% overlap in the cold-stress genes of the two varieties, and that K326-type *N*. *tabacum*, with better cold resistance, had more significant differential gene expression; plus, energy metabolism and hormone metabolism had obvious functions in the two ecotypes [6]. Current research is mainly focused on the low-temperature or cold resistance of model plants, crops and cash crops, but there have been few reports on the cold-resistance mechanism in ornamental garden plants.

Members of the family Liliaceae, including *Hosta* species, are rich in germplasm resources in terms of leaf type, flower color, and mosaic pattern. They are one of the most popular ornamental plants and are widely used in landscaping in many countries. In addition, the tender leaves of many *Hosta* species can be eaten, and they have some anti-inflammatory and analgesic effects. Many secondary metabolites have been demonstrated to have beneficial medicinal effects [12], but these *Hosta* species have poor cold resistance. None of the introduced species in Northeast China can cope with extreme low-temperature conditions, often causing death. The most remarkable feature of *Hosta ventricosa* is that it can tolerate extreme cold in winter. It is one of the few *Hosta* species that can survive the winter without protection in the northeast. It combines a high ornamental value with the advantages of cold resistance, shade tolerance and disease resistance, and it is the most widely used ground cover plant for urban garden landscaping in Northeast China. To date, there have been only a few studies on the mechanism of low-temperature resistance in *Hosta* species, which have mainly focused on physiological changes and altered gene expression. For example, treatment of *H*. *capitata* with antifreeze protein (AFP) at a concentration of 100 μg/L can effectively protect this plant species from damage caused by a temperature of 4°C, and reduces the expression of *CBF1* (C-crpeat binging transcription factor1) and *DHN1* (Dehydrin1) and increases the expression of SOD and CAT [13]. However, as yet there have been no published reports on the use of transcriptomes and metabolomes to conduct in-depth research on the cold-resistance mechanism of *H*. *ventricosa*. The findings of such a study would not only improve understanding of the mediation of this mechanism in this species, but might also form the basis for the development and identification of new varieties of *Hosta* with low-temperature resistance.

## Materials and methods

### Test materials and pretreatment

*Hosta ventricosa* that had been planted in the horticulture field of Jilin Agricultural Science and Technology College was transplanted into 21-cm diameter flower pots in May 2019. After three months of shaded and slow seedling cultivation, the potted plants were placed indoors. The test seedlings were divided into two groups. The no low-temperature treatment group (NT) was kept under normal indoor maintenance conditions and management regime at 25°C, and the low-treatment group (LT) was placed in an artificial climate box for low-temperature stress treatment. The final low-temperature treatment was set to 3°C [14]. In order to avoid large temperature changes causing tissue damage, the temperature was gradually decreased in 5°C increments, each lasting for 3 days, until the temperature of the climate chamber reached 3°C. The stress treatment was applied at 3°C for 7 days, and for each treatment group there were three replicates. The above-ground parts of the plants in the two test groups were removed, and the roots were collected. The roots were washed with double-distilled water and then immediately frozen in liquid nitrogen and stored in a freezer at −80°C.

### Transcriptomics analysis

#### RNA extraction

Total RNA was extracted from the plant roots using a Total RNA Purification Kit, TRK1001 (LC Science, Houston, TX), and was then subjected to agarose gel electrophoresis. The quality of DNA samples was checked using a NanoDrop 2000 spectrophotometer. The A260/280 quality was in the range of 1.70–1.90 and the concentration was 500 μg L^−1^ or more, meeting the requirements of subsequent tests. The samples were stored in a freezer at −80°C.

#### Library construction, sequencing and annotation

The sequencing work was undertaken by Hangzhou Lianchuan Bio Technologies Co., Ltd., and the NovaSeq 6000 System (Illumina, USA) was used for sequencing. In order to maximize the data quality, the original data were filtered, and Cutadapt and perl scripts were used to delete the contaminated, low-quality and undetermined bases. The sequence quality, including effective sequencing quantity, Q20, q30 and GC content, was estimated using FastQC (http://www.bioinformatics.babraham.ac.uk/projects/fastqc/). The transcriptome was then reassembled using Trinity 2.4.0 to obtain Unigene. DIAMOND was used to add the corresponding functional annotations to the assembled Unigene, and to select Nr (http://www.ncbi.nlm.nih.gov/), GO (http://www.geneontology.org), SwissProt (http://www.expasy.ch/sprot/), KEGG (http://www.genome.jp/kegg/), Pfam (http://pfam.xfam.org/) and EggNog (http://eggnogdb.embl.de/).

#### Screening of differentially expressed genes

Salmon [15] quantified the expression level of Unigenes by calculating TPM [16]. The differentially expressed Unigene was selected by the R package edge R [17], and the screening criteria were log2 > 1 or log2 < −1 (*P* < 0.05).

#### Fluorescence-based quantitative PCR

The transcription levels of differentially expressed genes (DEGs) in *H*. *ventricosa* were determined by quantitative real-time PCR (qRT PCR), which was performed using SYBR Premix Ex Taq (Tli Rnase H Plus, Takara). cDNA (20 times diluted) was used as the template for qRT PCR. The operation process and reaction conditions were as described in the manufacturer’s instructions, and the qRT PCR was repeated three times. The relative gene expression was calculated using the comparative 2^-ΔΔCT^ method. The gene-specific primers for qRT PCR were designed using Primer Express Software v3.0, and the *Hosta* Actin gene was used as the internal reference gene. The qRT PCR specific primers and internal reference primers are listed in Table 1.

**Table 1:** Primers of the qRT-PCR verification test.

Primer name	Sequence (5’→ 3’)	Primer name	Sequence (5’→ 3’)
HvActin-F	AACCACCTTAATCTTCATGCTGCT	HvActin-R	AAGATTCAGATGCCCAGAGGTCCT
DN34853 c1 g2-F	CGTTTCAAGCAGTTCGCTCTAC	DN34853 c1 g2-R	ATCCACCTGCCCGTCTGTATTG
DN32930 c0 g1-F	ATCCTCGTACAAGATTGACTGC	DN32930 c0 g1-R	TTCGCAAGAGCCTGTCTCATTT
DN35129 c1 g2-F	GGATGAATCAGAGCAGGGAATA	DN35129 c1 g2-R	GAATCAAGAGGTTTATCAGGAGC
DN30894 c0 g4-F	GCAGGTAGTTCTGTGGGTAGCA	DN30894 c0 g4-R	GATGGTCCCAAAGATGTAGCC
DN29020 c0 g1-F	AGACCCAGGAGGAGACGAGA	DN29020 c0 g1-R	TGTCCGCTGTCTCACGCCA
DN30502 c0 g4-F	GTATGGTCCTTCTGCACGAGTC	DN30502 c0 g4-R	TTGTTCAGTGTTAGACGGAGGA
DN31874 c1 g3-F	CACTTTGACACTGCTTCCCTGTA	DN31874 c1 g3-R	CTGGAAGGACAAGATGAGGGTG
DN35174 c1 g1-F	AAGGCTATTCACGGAGGCAACT	DN35174 c1 g1-R	AGCTCGGAGAACTGAGCAAACAT
DN30864 c1 g1-F	ATGCTCCTCCTCCCAAACTCC	DN30864 c1 g1-R	TCCTTGACTTTGCTGGCGTA
DN24297 c3 g2-F	GATGAACTTCTTCGGCAACC	DN24297 c3 g2-R	CTCCTCACCGACGCCTGTTT
DN27133 c0 g2-F	GACGAGCACATGGCGAAC	DN27133 c0 g2-R	CGGTTCCACCAAACATCACAT
DN29325 c0 g1-F	GGTTTCTTTCCTTGGGTTCG	DN29325 c0 g1-R	TGAAAGACGCAGCGGCA
DN26665 c0 g6-F	AGTCCTGCCGAAAGAAGTGG	DN26665 c0 g6-R	AAGAGGAGCACCATTCCAGTC
DN33602 c0 g1-F	CGGCGGTTACGACATTCCTG	DN33602 c0 g1-R	CTTGAAATCGTTGCCGTTGG

### Metabolomics analysis

#### Extraction of samples

The root samples that had been prepared were vacuum freeze-dried, the dried sample was ground to a powder, and 100 mg of the powder was then dissolved in 0.6 mL of 70% methanol extract, stored at 4°C for 12 h, and vortexed six times during this period to improve the extraction efficiency. The extract was centrifuged (rotating speed: 10000 g for 10 min), and the supernatant was then collected, passed through a 0.22-μm filter membrane, and stored in a sample bottle for UPLC-MS/MS analysis.

#### Ultra-performance liquid chromatography (UPLC)

The sample extracts were analyzed using an UPLC-ESI-MS/MS system (UPLC, Shim-pack UFLC SHIMADZU CBM30A system, www.shimadzu.com.cn/; MS, Applied Biosystems 4500 Q TRAP, www.appliedbiosystems.com.cn/). The analytical conditions were as follows: UPLC: column, Agilent SB-C18 (1.8 μm, 2.1 mm × 100 mm); the mobile phase consisted of solvent A, pure water with 0.1% formic acid, and solvent B, acetonitrile. Sample measurements were performed with a gradient program that employed the starting conditions of 95% A and 5% B. Within 9 min, a linear gradient to 5% A and 95% B was programmed, and a composition of 5% A and 95% B was kept for 1 min. Subsequently, a composition of 95% A and 5.0% B was adjusted within 1.10 min and kept for 2.9 min. The column oven was set to 40°C and the injection volume was 4 μL. The effluent was alternatively connected to an ESI-triple quadrupole-linear ion trap (QTRAP)-MS.

#### Qualitative and quantitative analysis of metabolites

A self-built MetWare database was used for qualitative analysis of metabolites based on secondary spectral data. During analysis, the isotope signals were removed, including repeated signals of K^+^, Na^+^ and NH_4_^+^, as well as repeated signals of fragment ions of other higher-molecular-weight compounds. The quantitative analysis of metabolites was accomplished using multiple reaction monitoring analysis of triple quadrupole mass spectrometry. After the metabolite spectrum analysis data had been obtained from all the samples, the area of the mass spectrum peaks of all metabolites was integrated, and the mass spectrum peaks of the same metabolite in different samples were integrated and corrected [18].

#### Differential metabolites selected

Significantly regulated metabolites between groups were determined by VIP ≥ 1 and absolute Log2FC (fold change) ≥ 1. VIP values were extracted from the OPLS-DA result, which also contained score plots and permutation plots, and was generated using the R package MetaboAnalystR. The data were log transformed (log2) and mean centered before OPLS-DA. In order to avoid overfitting, a permutation test (200 permutations) was performed.

#### KEGG annotation and enrichment analysis

Identified metabolites were annotated using the KEGG Compound database (http://www.kegg.jp/kegg/compound/), and then annotated metabolites were mapped to the KEGG Pathway database (http://www.kegg.jp/kegg/pathway.html). Pathways with significantly regulated metabolites mapped to them were then fed into MSEA (metabolite sets enrichment analysis), and their significance was determined by hypergeometric test *p*-values.

## Results and analysis

### Transcriptome analysis

#### Gene assembly, annotation and statistics of the number of differences

A total of six cDNA libraries of purple calyx hosta (*H*. *ventricosa*) were sequenced using the Illumina HiSeq6000 platform. After filtering and assembling the original data, a total of 64 848 genes were obtained, with a GC content of 42.91%. The shortest gene sequence was 201 bp and the longest was 13 478 bp, with an average length of 372 bp and an N50 length of 1264 bp, indicating that the sequencing quality was satisfactory. Requirements can be used for subsequent analysis. The 64 848 genes obtained included 22 210 GO annotations (34.25%), 17 924 KEGG annotations (27.64%), 19 634 Pfam annotations (30.28%), 18 237 SwissProt annotations (28.12%), 26 067 EggNOG annotations (40.20%) and 28 479 NR annotations (43.92%). A total of 12 059 DEGs were obtained, of which 6737 genes were up-regulated and 5322 genes were down-regulated. The results are shown in Table 2.

**Table 2:** Transcriptome numbers.

Index	All	GC%	Min length	Median length	Max length	Total assembled bases	N50
Transcript	162626	43.50	201	639.00	13478	151507228	1465
Gene	64848	42.91	201	372.00	13478	46504372	1264
DB	All	GO	KEGG	Pfam	swissprot	eggNOG	NR
Num	64848	22210	17924	19634	18237	26067	28479
Ratio (%)	100%	34.25	27.64	30.28	28.12	40.20	43.92
All DEGs	12059	Up DEGs	6737	Down DEGs	5322		

#### Verification of DEGs by qRT PCR

**Fig 1:**
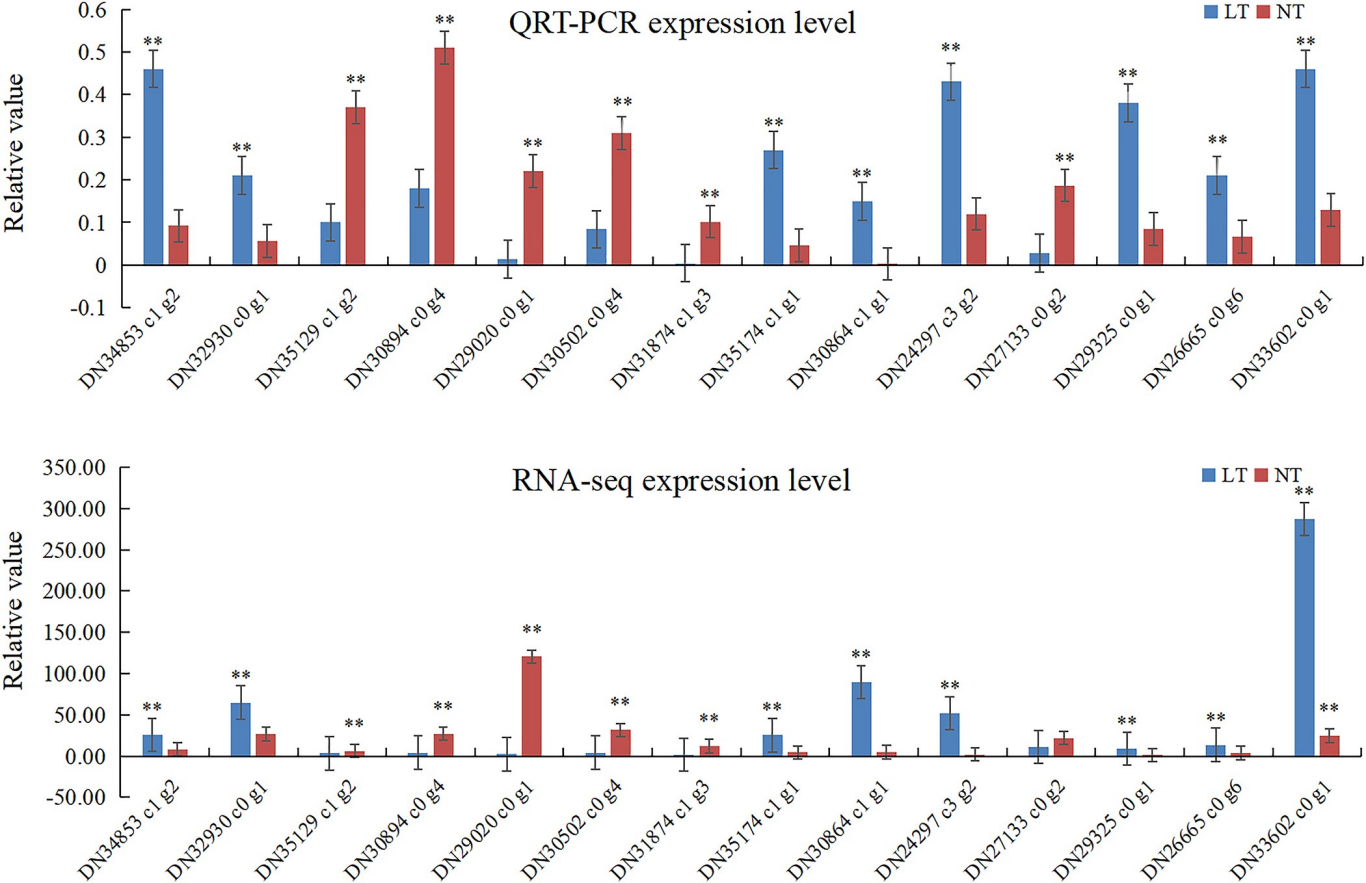
The 14 differentially expressed genes that were verified by qRT PCR.

In order to verify the reliability and reproducibility of RNA-Seq, 14 DEGs from *H*. *ventricosa* were randomly selected for qRT PCR to analyze their expression under low-temperature stress (Fig 1). These genes included functional proteins and transporters, protective enzymes and transcription factors. The expression of 13 diferential genes was consistent with that of RNASeq, with the exception of DN27133 c0 g2, which indicated that the sequencing results were reliable. The difference between LT and NT is shown in Fig 1 (** represents P<0.01).

#### GO and KEGG enrichment analysis of DEGs

**Fig 2:**
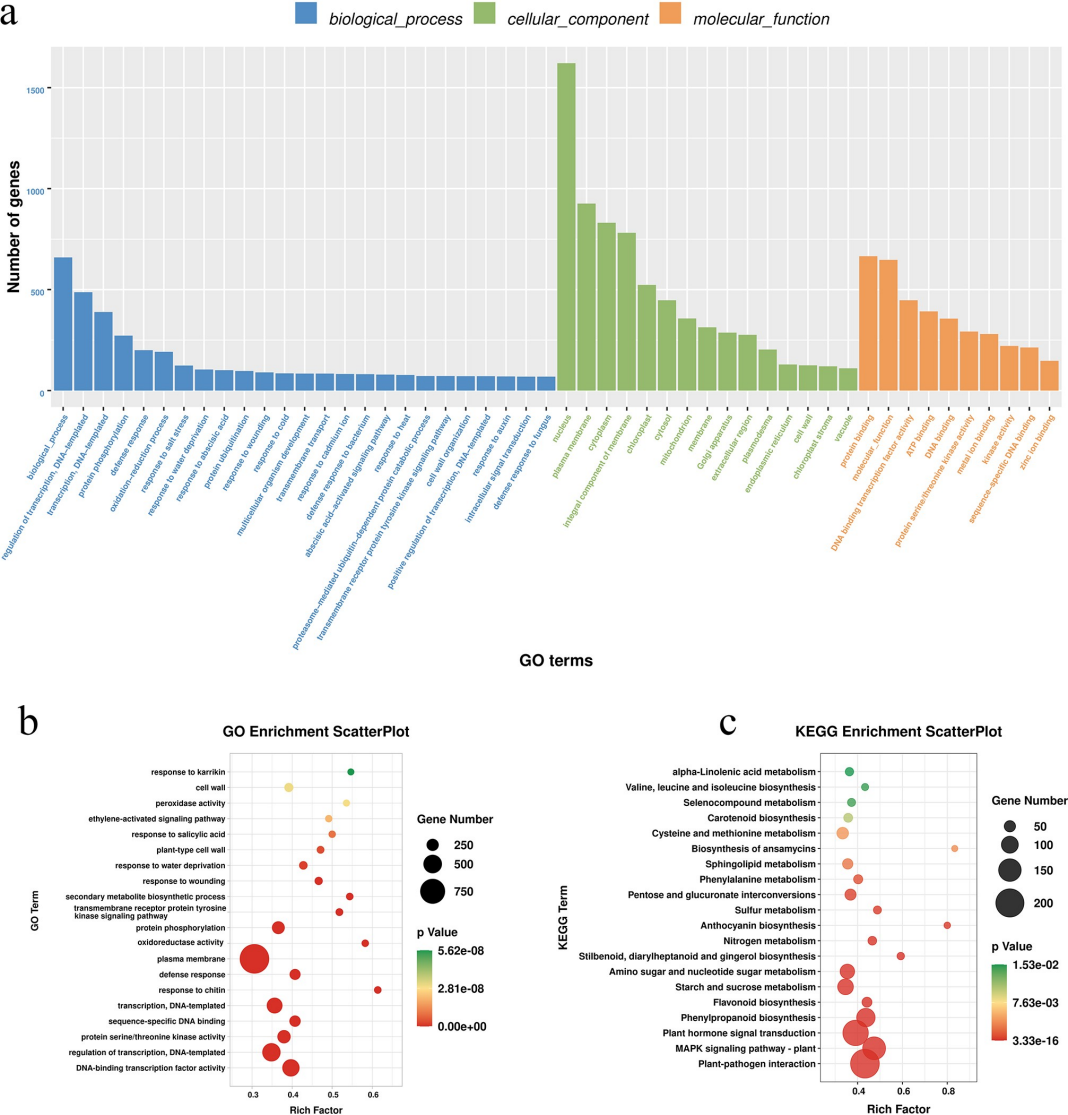
GO and KEGG enrichment analysis of differentially expressed genes. a. Differential gene composition structure based on GO. b. Differential gene GO enrichment items. c. Differential gene KEGG enrichment items.

GO analysis was used to classify 3215 differential gene functions (Fig 2A), 54.57% of which were annotated to "biological process", 12.18% to "cellular component" and 33.25% to "molecular function". The differences between genes annotated in GO and KEGG were analyzed for significance. Fig 2B and 2C show the results for the top 20 *P*-values only. There were 300 distinct GO classification enrichment sub-items in *H*. *ventricosa*, which were mainly associated with the plasma membrane, defense response, transcription, and protein serine or threonine kinase activity. A total of 28 distinct KEGG-associated pathways were obtained from *H*. *ventricosa* root tissue, which were mainly associated with plant–pathogen interactions, the MAPK signal transduction pathway, plant hormone signal transduction, phenylpropanoid biosynthesis and flavonoid biosynthesis.

### Metabolomics analysis

#### Differential metabolite analysis

**Fig 3:**
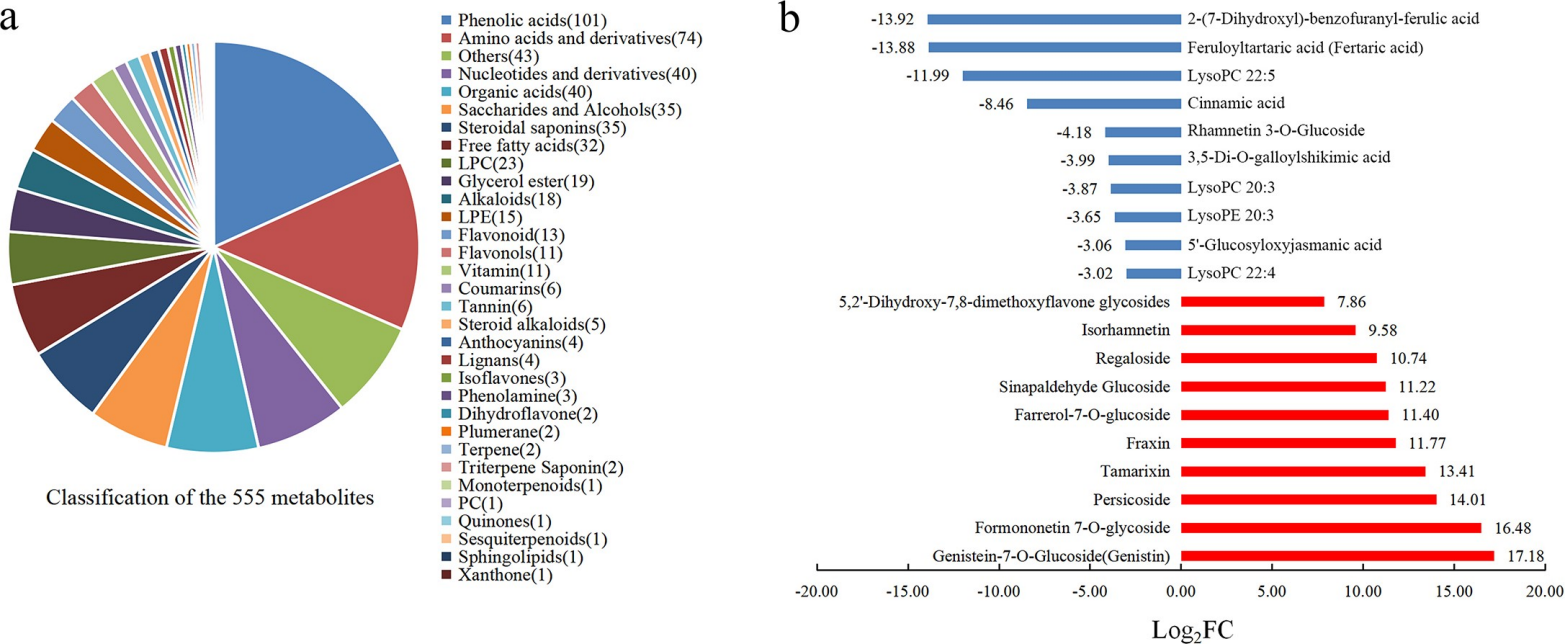
Metabolite analysis. a. Classification of metabolites. b. Significantly different metabolites (only the top 20 *P*-values are shown; blue bars denote down-regulated expression, and red bars denote up-regulated expression).

A total of 555 metabolites were detected in the root system. These metabolites were divided into 32 categories, including phenolic acids (101 metabolites), amino acids and derivatives (74 metabolites), nucleotides and derivatives (40 metabolites), and organic acids (40 metabolites) (see Fig 3A for details). Comparing the fold changes in the quantitative data for metabolites in the LT and NT groups, 131 of the 555 metabolites showed significant differences, including genistein-7-O-glucoside (Genistin) and formononetin 7-O-glycoside. Comparing LT and NT, a total of 77 up-regulated metabolites, such as persicoside, as well as 54 down-regulated metabolites, such as 2-(7-dihydroxyl)-benzofuranyl-ferulic acid, feruloyltartaric acid (Fertaric acid) and LysoPC 22: 5, showed significant changes. The results of the metabolite analysis are presented in Fig 3B.

#### Functional annotation and enrichment analysis of differential metabolite KEGG

**Fig 4:**
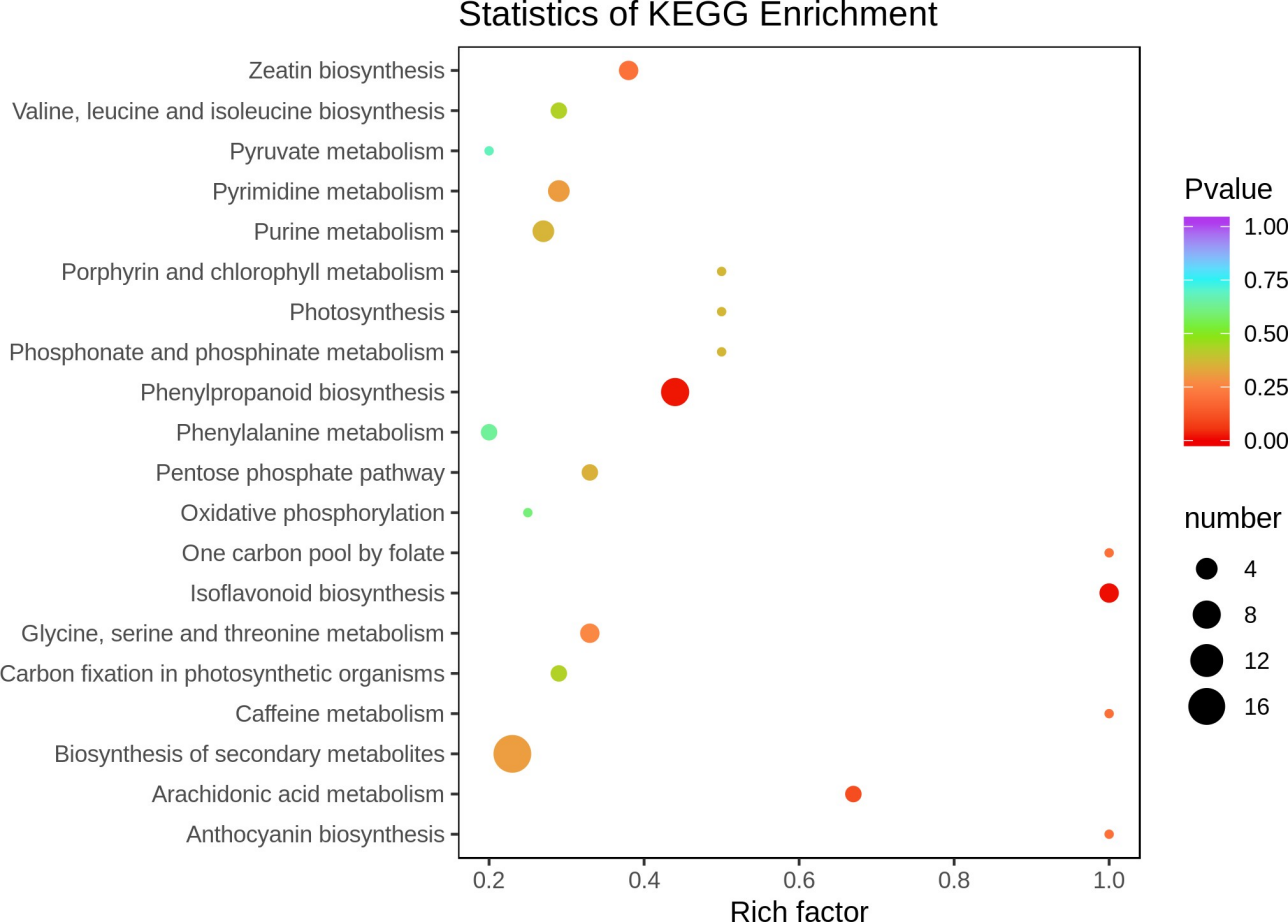
Data for enrichment of significantly different metabolites (only the top 20 pathways are shown).

The significantly different metabolites were enriched according to the type of KEGG pathway. The *P*-value and Rich factor were used as the screening conditions, and the top 20 metabolites with significant differences and higher enrichment coefficients were plotted. The results are shown in Fig 4. Isoflavonoid biosynthesis had a high enrichment coefficient, significant difference and number of differences. Although phenylpropanoid biosynthesis had a low enrichment coefficient, the difference was significant. This indicates that the metabolic pathways of isoflavonoid biosynthesis and phenylpropanoid biosynthesis play an important role in the low-temperature resistance of the *H*. *ventricosa*.

### Correlation analysis between transcriptomes and metabolomes

**Fig 5:**
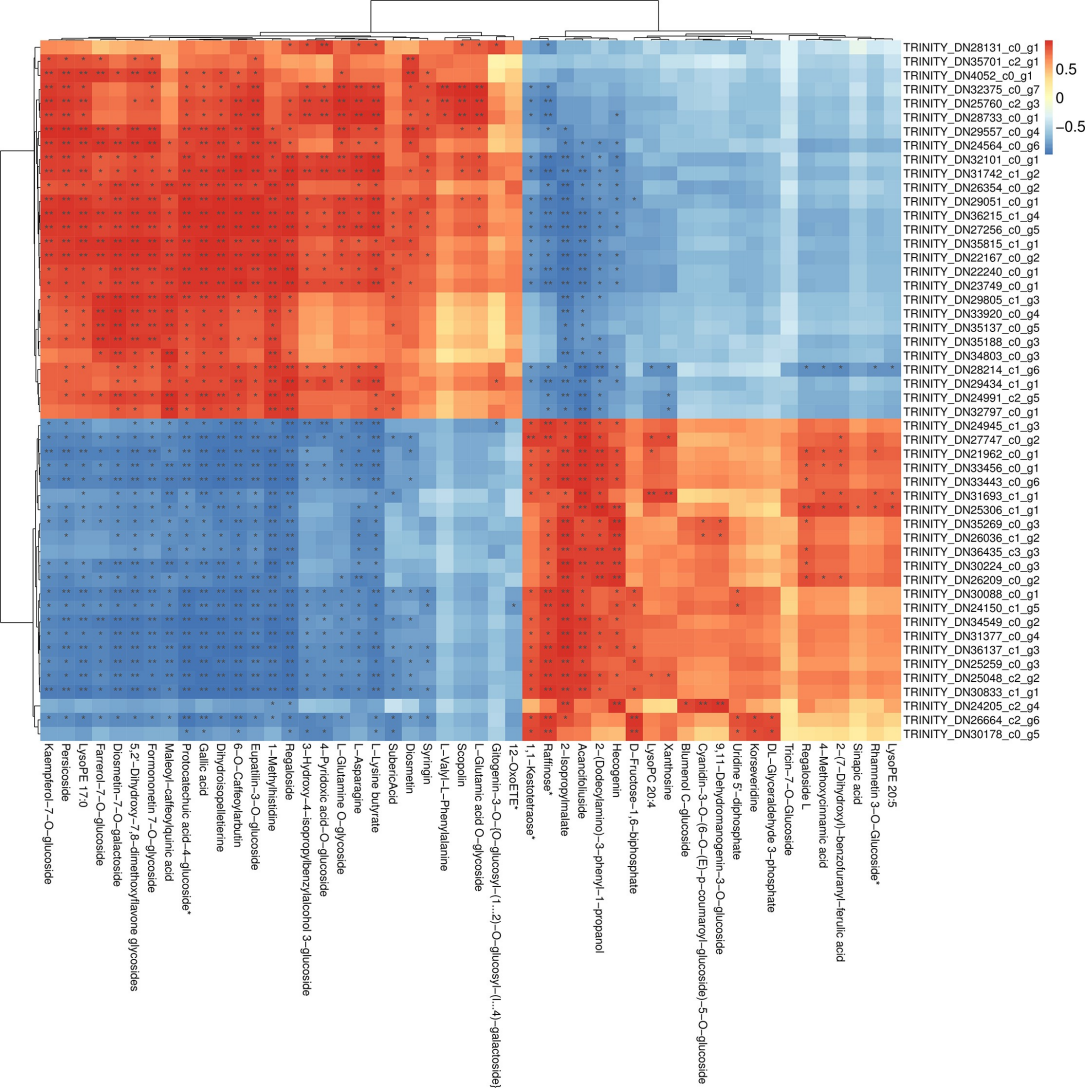
Correlation heat map analysis of transcriptome and metabolome.

Use a correlation absolute value of >0.9 and *p*-value of <0.01 as the screening conditions to draw a heat map (Fig 5), the horizontal axis represents metabolites, the vertical axis represents genes, red represents positive correlation between genes and metabolites, and blue represents genes and metabolism. As can be seen, there is a negative correlation between objects. The darker the color, the stronger the correlation. A single asterisk (*)in the figure represents a *p*-value <0.05 and a double asterisk (**) a *p*-value <0.01. For example, scopoline has a strong correlation with TRINITY_DN32375_C0_G7, TRINITY_DN25760_C2_g3 and the difference is extremely significant. The correlation between the remaining differential metabolites and the differential gene is shown in Fig 5.

## Result and discussion

The introduction and domestication of ornamental plant species is mainly affected by climate, especially in seasonally frozen regions at mid and high latitudes. The long cold winters in Northeast China severely restrict the range of ornamental plants that can be introduced. Many fine ornamental plants have failed to domesticate owing to climate inadaptability. *Hosta ventricosa* is widely used for planting under forest in Jilin Province because of its excellent low-temperature tolerance. To date, studies on low-temperature resistance in *Hosta* species have been limited to the exogenous application of AFPs to protect *H*. *capitata* from cold stress [13], and the majority of the research on low-temperature resistance has focused on rice [19], cabbage [8] and other annual crops or model plants. Therefore, the investigation of low-temperature resistance in *H*. *ventricosa* may not only identify the mechanism of such resistance in this species, but also form the basis for the development of new varieties of *Hosta* that can tolerate low temperatures. Transcriptomic and metabolomic technologies are often used to identify regulatory mechanisms, to explore the growth, development, and physiological and pathological response mechanisms of organisms, and by combining transcriptomics and metabolomics through the KEGG metabolic pathway to identify genes and metabolites involved in the same biological process. Significant changes in genes and metabolites are a very effective method.

### Transmission of low-temperature signals in *H*. *ventricosa*

The cold response and cold defense mechanisms of plants are highly complex, involving multiple synergistic signal transduction and metabolic pathways, such as ROS protection, Ca^2+^ signal transduction, and changes in cell membrane structure and components [7]. In the present study, a total of 12 059 DEGs were obtained, of which 3188 DEGs were annotated to 131 KEGG pathways, and these differential genes were mainly concentrated in plant–pathogen interactions and signal transduction (MAPK signaling pathways). The DEGs in the four pathways of plant hormone signal transduction and phenylpropanoid biosynthesis accounted for 20.36% of the DEGs that had been annotated on KEGG. Plants perceive cold stimuli through changes in the plasma membrane. Low temperature affects the composition and structure of the cell membrane, triggering the process whereby Ca^2+^ enters the cell via ion channels on the cell membrane, and activating the cell’s defense or immune response through Ca^2+^ signaling.

In this study, it was found that at least three channel proteins—namely, COLD, CNGCs and CRLK—interact with Ca^2+^ to transmit cold signals across the root cell membrane in *H*. *ventricosa*. COLD1 is a cold-sensing signal sensor found in rice. It can recognize changes in cell membranes to sense cold signals, and activate Ca^2+^ channels through the action of the G protein α subunit. Rice (*Oryza sativa*) that lacks *COLD1* is more sensitive to cold. Furthermore, overexpression significantly improves the cold tolerance of rice [20]. There is only one gene annotated as *COLD1* in *H*. *ventricosa*, and its expression is significantly up-regulated under cold stress. It is speculated that in this species *COLD1* may have a similar function to *OsCOLD1*, and may play an important role in sensing low temperature during periods of low-temperature stress. CNGCs are another type of non-selective cation channel located on the cell membrane, and have been shown to be related to a variety of external stimuli and to influence plant immune activities. *OsCNGC6* and *OsCNGC16* in rice can be significantly induced at low temperatures [21]. There is also significant differential expression of different types of CNGCs in the root system of *H*. *ventricosa*. This includes the up-regulated expression of *CNGC4*, *CNGC5*, *CNGC20* and *CNGC2*, and the down-regulated expression of *CNGC15C*. These CNGCs may participate in the cold mediation process. However, a study of CNGCs in mustard found that they are also responsible for the regulation of salt, heavy metals, germs, and growth processes [22], so the role of CNGCs in the regulatory mechanism of *H*. *ventricosa* may be more complex. CRLK1 has two calmodulin-binding sites with different affinities, which can sense cold and hydrogen peroxide. In *A*. *thaliana*, CRLK1 knockout mutant plants exhibit significant low-temperature sensitivity [23]. In *H*. *ventricosa*, two genes have been identified—namely, *CRLK1* and *CRLK2*—both of which are up-regulated. This finding is consistent with the results reported for *OsCRLK1* in rice [24].

Although there are multiple low-temperature-related ion channels or sensors on the cell membrane of *Hosta*, they all interact with Ca^2+^ to transmit low-temperature signals. In many studies pointed out that the MAPK signaling pathway is one of the important signal mechanism in plants, this pathway can be activated by a variety of signals, such as hydrogen peroxide, abscisic acid (ABA), ethylene, low temperature or salt, and then through gradual protein phosphorylation, it triggers the changes of transcription factor, phospholipase and other proteins’ activity, or phosphorylation of specific targets in response to environmental stimuli [25,26]. Studies have shown that MAPK is involved in the conduction of signals related to external stimuli such as non-essential heavy metals [27], drought [28], low temperature [29] and pathogenic injury [30]. A large number of DEGs have been identified in the MAPK pathway in *H*. *ventricosa*. Ca^2+^ signaling positively regulates the MAPK-mediated plant response to cold stress through CPKs. In *H*. *ventricosa*, *CPK17* is up-regulated and expressed, triggering MEKK1-MKK3-MPK4. In the cascade of 6, *MKK3* and *MPK4/6* are up-regulated, but the expression of *MEKK1* remains unchanged. It has also been reported that MEKK1 can be phosphorylated by CRLK1 [31].

CBL is a Ca^2+^ signal sensor. It interacts with CIPK to relay cold-triggered Ca^2+^ signals into phosphorylation [32]. In *H*. *ventricosa*, *CBL1* significantly up-regulates the expression, but its associated *CIPK*s expression is consistent. In rice and *Arabidopsis*, *CIPK1* and *CIPK7* are induced by low temperatures. Research on the effect of low temperature on cassava (*Manihot esculenta*) also showed that *CIPK10* is induced by cold stress in the root system. These findings are similar to the *CBL*-*CIPK* effect in *H*. *ventricosa* [33–35]. In addition, studies have shown that both MAPKs and *CBL*-*CIPK* can interact with *ICE1* to bind to *CBF* or *DREB* and trigger plant cold defense responses [36]. *Arabidopsis ICE1* has been confirmed to be a cold-mediating gene, and overexpressed *ICE1* can interact with *CBF3*/*DREB1A* to enhance the freezing resistance in this genus [37]. *Hosta ventricosa SCRM* is an *ICE1*-like transcription factor. In the present study, *SCRM* had the same effect as *ICE1*, and mediated *H*. *ventricosa DREB1A* under conditions of cold stress. However, transcriptome analysis has shown that there are many members of the *DREB* family, including *DREB2A*, *DREB1B*, *DREB2B*, *DREB1D*, *DREB1E* and *DREB1F*, among others. Although these genes are similar to *DREB1A* and are all down-regulated, and it is unclear whether they are affected by other functions. In addition, there have been many reports that *CAMTA3* is a calmodulin transcription factor, and *Arabidopsis* knockout mutants show higher sensitivity to low temperatures. *CBF1* can also recognize *CAMTA3* [37], but this study did not find *CAMTA3* in *H*. *ventricosa*, which may require more experiments to verify.

In addition, hydrogen peroxide, ethylene, abscisic acid and jasmonic acid are involved in the signaling and defense responses of plants to low temperatures. Combined with the determination of plant hormones, more important information may be obtained.

### Involvement of the phenylpropanoid and flavonoid metabolic pathways in the cold defense response of *H*. *ventricosa*

The transcriptomics and metabolomics of *H*. *ventricosa* show that a large number of DEGs and metabolites are enriched in the metabolic pathways of phenylpropanoid and flavonoid biosynthesis. Many studies have shown that the phenylpropanoid biosynthetic pathway is affected by drought, heavy metals, temperature and other abiotic stresses. Its activation leads to the accumulation of a range of phenolic compounds, which include metabolites such as phenolic acid and flavonoids. During these biological processes, multiple genes are involved in the synthesis of metabolites [38]. In the leaves of maize (*Zea mays*), the levels of phenolic compounds and anthocyanins increase as the temperature decreases, and there is an increase in the expression of genes that encode enzymes of the phenylpropanoid pathway, which in turn results in increased activity of cinnamate-4-hydroxylase (C4H) and chalcone synthase (CHS) [39]. When *Fagopyrum tataricum* is subjected to low-temperature treatment, most of the transcripts in the phenylpropanoid biosynthetic pathway are up-regulated, and the levels of anthocyanin and proanthocyanidin increase significantly [40]. In cold-acclimated tobacco (*Nicotiana tabacum*) it has been demonstrated that a number of DEGs and differential metabolites that have a role in signal transduction, carbohydrate metabolism and phenylpropanoid biosynthesis are involved in the low-temperature defense process [41].

Metabolome analysis of *H*. *ventricosa* identified 32 differentially expressed phenolic compounds, including cinnamic acid, the first intermediate in the pathway of phenylpropanoid biosynthesis. In that metabolic pathway, PAL catalyzes the conversion of phenylalanine to trans-cinnamic acid (t-CA), which is the precursor for many active metabolites. However, cinnamic acid has two isomers in plants—namely, cis-cinnamic acid (c-CA) and trans-cinnamic acid (t-CA). Only t-CA enters the phenylpropanoid pathway. c-CA may be an ultraviolet-light-mediated t-CA isomerization product, which has a role in regulation of the dynamic balance of auxin [42]. The UPLC-MS/MS technology that was used in the present study cannot distinguish between isomers, so it is assumed that the cinnamic acid involved in the phenylpropanoid biosynthetic pathway is t-CA.

Phenylpropionic acid in *H*. *ventricosa* is catalyzed by the up-regulated expression of *PAL* (*PAL1*, *PAL2*, *PAL3*) to form t-Ca which forms P-coumaroyl-CoA under the action of *4Cl* (*4Cl1*, *4Cl2*) and cytochrome P450 (*CYP73A100*, *CYP73A13*), and p-Coumaroyl-CoA, as the core intermediate product of the phenylpropane metabolic pathway, can connect to the flavonoid metabolic pathway and part of it can form isoflavones, or other phenolic compounds formed by other means. Four significantly different metabolites were produced by the metabolic pathways of phenylpropanoid and flavonoid biosynthesis in *H*. *ventricosa*, including the two isoflavones formononetin 7-O-glucoside (FG) and genistein 7-O-glucoside (genistein), and the two phenolic compounds scopolin and syringin. There was also an increase in content of the precursors of phenolic compounds.

FG is a type of isoflavone that is commonly found in legumes. Many *in vitro* experiments have shown that FG has significant antioxidant and anti-inflammatory effects on other organisms [43], but there has been very little research on plant resistance. Some studies have shown that the FG content of red clover (*Trifolium pratense*) increases sharply after three weeks of submergence stress [44]. There is no direct evidence that FG is involved in plant cold stress, and FG can interact with plant hormones during plant growth. Leading to the decrease in plant hormones, it is speculated that the increase in FG content in *H*. *ventricosa* may be related to hormone cross-talk, which together affect the low-temperature resistance of this species [45]. Genistein is another type of isoflavone that is abundant in legumes, and it has a wide range of pharmacological properties. Many studies have reported that genistein can significantly improve the resistance of plants to biological stress [46]. For example, it has been shown to increase the resistance of alfalfa (*Medicago sativa*) to *Acyrthosiphon pisum* [47], and of rice (*O*. *sativa*) to *Magnaporthe oryzae* [48]. Genistein can also improve the response of plants to adverse environmental conditions. One study reported that after soybean (*Glycine max*) was subjected to low temperature (10°C) for 24 hours, the genistein content of the root system increased threefold compared with the untreated group [49]. The same conclusion was also reflected in the low-temperature osmotic compound stress of soybean [50]. There are also some studies which suggest that genistein is linked to high temperature stress. The genistein content of soybean (*G*. *max*) increases significantly with rising temperature, but it has also been noted that the genistein content of different soybean genotypes does not always increase in response to high-temperature stress [51]. Although these studies did not identify the mechanism underlying the response of genistein to temperature change, they did show that genistein content is closely related to temperature change, which is consistent with the finding that in *H*. *ventricosa* under low-temperature stress the genistein content increased significantly. The above-mentioned studies all used soybean as the experimental material. However, there are no reports on genistein content under abiotic stress in other crop plants, and further research is needed to clarify the mechanism underlying the response of genistein to changes in temperature.

Scopoletin is the precursor of scopolin, and both compounds are derivatives of coumarin. They can participate in the elimination of ROS and in defense against pathogens. The content of scopoletin and scopolin in *H*. *ventricosa* is significantly increased, which indicates that both compounds have a role in the low-temperature defense process in this species. Low-temperature stress can induce the accumulation of scopolin in *Arabidopsis*, and a decrease in scopolin accumulation is observed in *Arabidopsis* mutants that lack the genes related to scopolin production [52]. Under a combination of drought and high-temperature stress, the scopolin content of mandarin orange (*Citrus reticulata*) increases, enabling the plant to cope with these adverse environmental conditions. It has been suggested that scopolin reduces oxidative damage in citrus [53], grape (*Vitis vinifera*) [54] and tea tree (*Camellia sinensis*) [55].

Sinapyl alcohol is a constituent of lignin, which is a component of the cell wall that plays an important role in resisting abiotic stress [56]. Cinnamyl alcohol dehydrogenase (CAD), which has been identified in sweet potato (*Ipomoea batatas*), catalyzes the last step of the lignin synthesis pathway and is involved in the synthesis of lignin monomers. Overexpression of IbCAD1 in cassava can cause increased accumulation of sinapyl alcohol in the root system, and at the same time increases the plant’s tolerance of ROS such as hydrogen peroxide. Lignin is mainly composed of guaiacyl (G), syringyl (S) and p-hydroxyphenyl (H) units, which are derived from coniferyl alcohol, sinapyl alcohol and p-coumaryl alcohol, respectively [57]. The ratio between them can affect the adaptability of plants to environmental stress [58]. Under low-temperature conditions, the root system of *H*. *violacea* showed up-regulation of *PAL*, *4CL*, *F5H*, *CCOAOMT* and almost all other genes related to the synthesis of sinapyl alcohol via the phenylpropanoid biosynthesis pathway, whereas *CYP84A1*, *7OMT* and *CAD8* were all down-regulated. These genes promote a significant increase in the content of sinapyl alcohol. At the same time, the content of coniferyl alcohol in *H*. *ventricosa* is significantly reduced, which indicates that this species controls the composition of lignin by adjusting the G/S ratio, thereby altering the morphological structure of the cell wall.

## Conclusions

**Fig 6:**
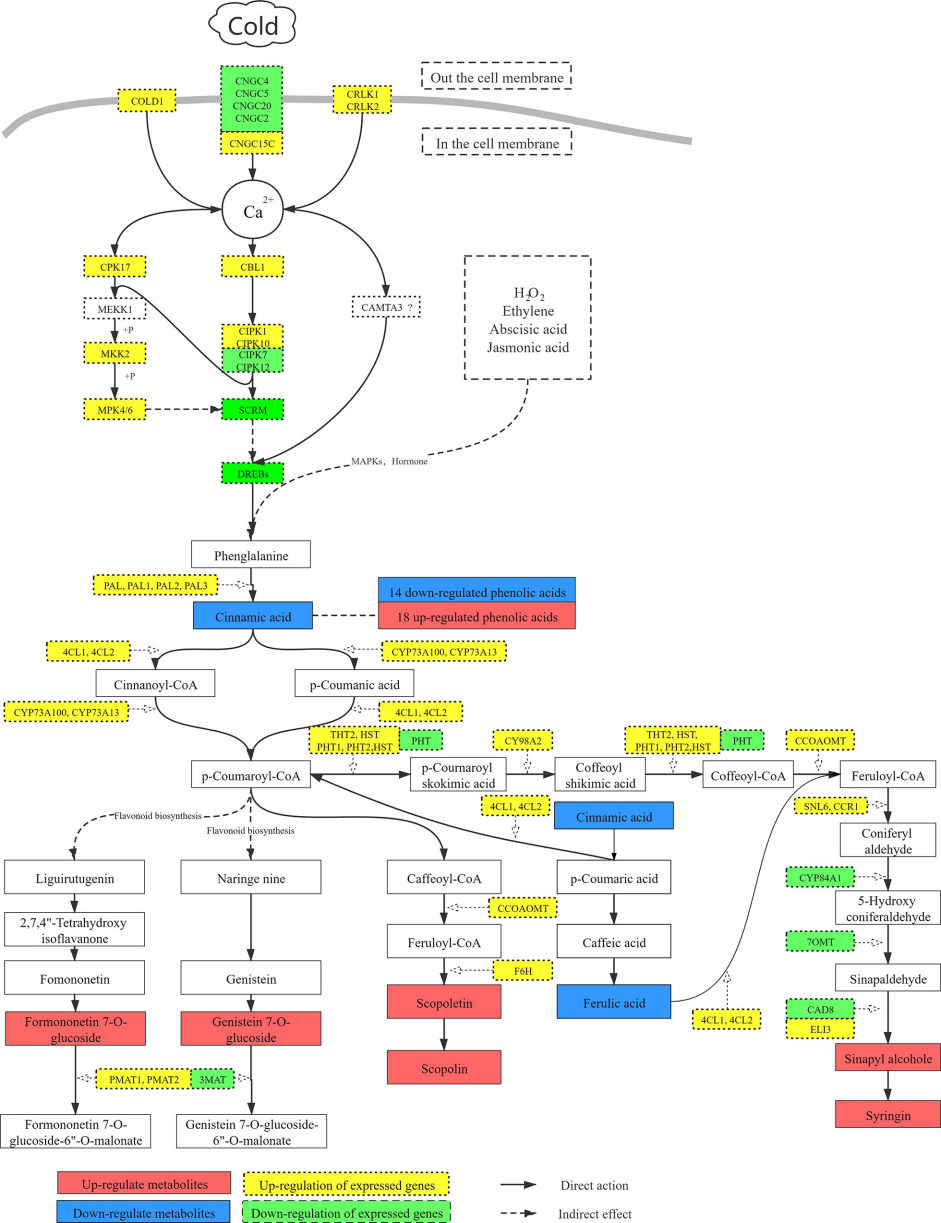
Signal transduction and the pathway of phenylpropanoid synthesis in *H*. *ventricosa*.

The majority of *Hosta* species are important ornamental plants worldwide, but many of them have low cold tolerance. This study used transcriptomics and metabolomics to investigate the potential impact of low-temperature stress on purple calyx hosta (*H*. *ventricosa*). Under low-temperature stress, 64 848 genes were identified in this species, and 12 059 DEGs were annotated, the latter being mainly associated with plant–pathogen interactions, the MAPK signal transduction pathway, plant hormone signal transduction, phenylpropanoid biosynthesis and flavonoid biosynthesis. Metabolomics analysis identified 555 metabolites, of which 131 metabolites showed significant differences in content, and were mainly enriched in the pathways of phenylpropanoid biosynthesis, isoflavonoid biosynthesis and arachidonic acid metabolism. With regard to low-temperature signal transduction, the transport of Ca^2+^ into cells in *H*. *ventricosa* occurs mainly through the ion channels in the cell membranes, involving COLD, CNGCs and CRLK. Ca^2+^ triggers the MAPK signal pathway and Ca^2+^ signal sensors such as CBL, and strengthens the low-temperature resistance of the *H*. *ventricosa* through the combination of *SCRM* and *DREB*. With regard to low-temperature defense responses, the genes and metabolites related to the pathways of phenylpropanoid metabolism and flavonoid metabolism are considered to be related to the low-temperature resistance of *H*. *ventricosa*. An increase in genistein, scopolentin and scopolin content possibly improves the plant’s low-temperature tolerance (Fig 6). The change in the content ratio of sinapyl alcohol and coniferyl alcohol affects the composition of lignin, which in turn influences the cell wall structure in *H*. *ventricosa*. However, further research is needed to clarify the role of FG in the plant response to abiotic stress, and to determine the relationship between endogenous hormones, low temperature and the expression of the relevant genes.
